# Blunt cerebrovascular trauma causing vertebral arteryd issection in combination with a laryngeal fracture: a case report

**DOI:** 10.1186/1752-1947-5-381

**Published:** 2011-08-15

**Authors:** Michael Frink, Carl Haasper, Kristina Imeen Ringe, Christian Krettek, Frank Hildebrand

**Affiliations:** 1 Trauma Department, Hannover Medical School, Carl-Neuberg-Str. 1, 30625 Hannover, Germany; 2 Institute of Radiology, Hannover Medical School, Carl-Neuberg-Str. 1, 30625 Hannover, Germany

## Abstract

**Introduction:**

The diagnosis and therapy of blunt cerebrovascular injuries has become a focus since improved imaging technology allows adequate description of the injury. Although it represents a rare injury the long-term complications can be fatal but mostly prevented by adequate treatment.

**Case presentation:**

A 33-year-old Caucasian man fell down a 7-meter scarp after losing control of his quad bike in a remote area. Since endotracheal intubation was unsuccessfully attempted due to the severe cervical swelling as well as oral bleeding an emergency tracheotomy was performed on scene. He was hemodynamically unstable despite fluid resuscitation and intravenous therapy with vasopressors and was transported by a helicopter to our trauma center. He had a stable fracture of the arch of the seventh cervical vertebra and fractures of the transverse processes of C5-C7 with involvement of the lateral wall of the transverse foramen. An abort of the left vertebral artery signal at the first thoracic vertebrae with massive hemorrhage as well as a laryngeal fracture was also detected. Further imaging showed retrograde filling of the left vertebral artery at C5 distal of the described abort. After stabilization and reconfirmation of intracranial perfusion during the clinical course weaning was started. At the time of discharge, he was aware and was able to move all extremities.

**Conclusion:**

We report a rare case of a patient with vertebral artery dissection in combination with a laryngeal fracture after blunt trauma. Thorough diagnostic and frequent reassessments are recommended. Most patients can be managed with conservative treatment.

## Introduction

Blunt cerebrovascular trauma is a rare entity and mostly caused by high energy accidents. Due to improved imaging of trauma patients the diagnosis can be made early while in the past most cases were diagnosed after patients were symptomatic. Transection as the most severe entity of vertebral artery injury is usually fatal [1]. We present the case of a patient with an isolated blunt craniocervical injury.

## Case presentation

**Figure 1:**
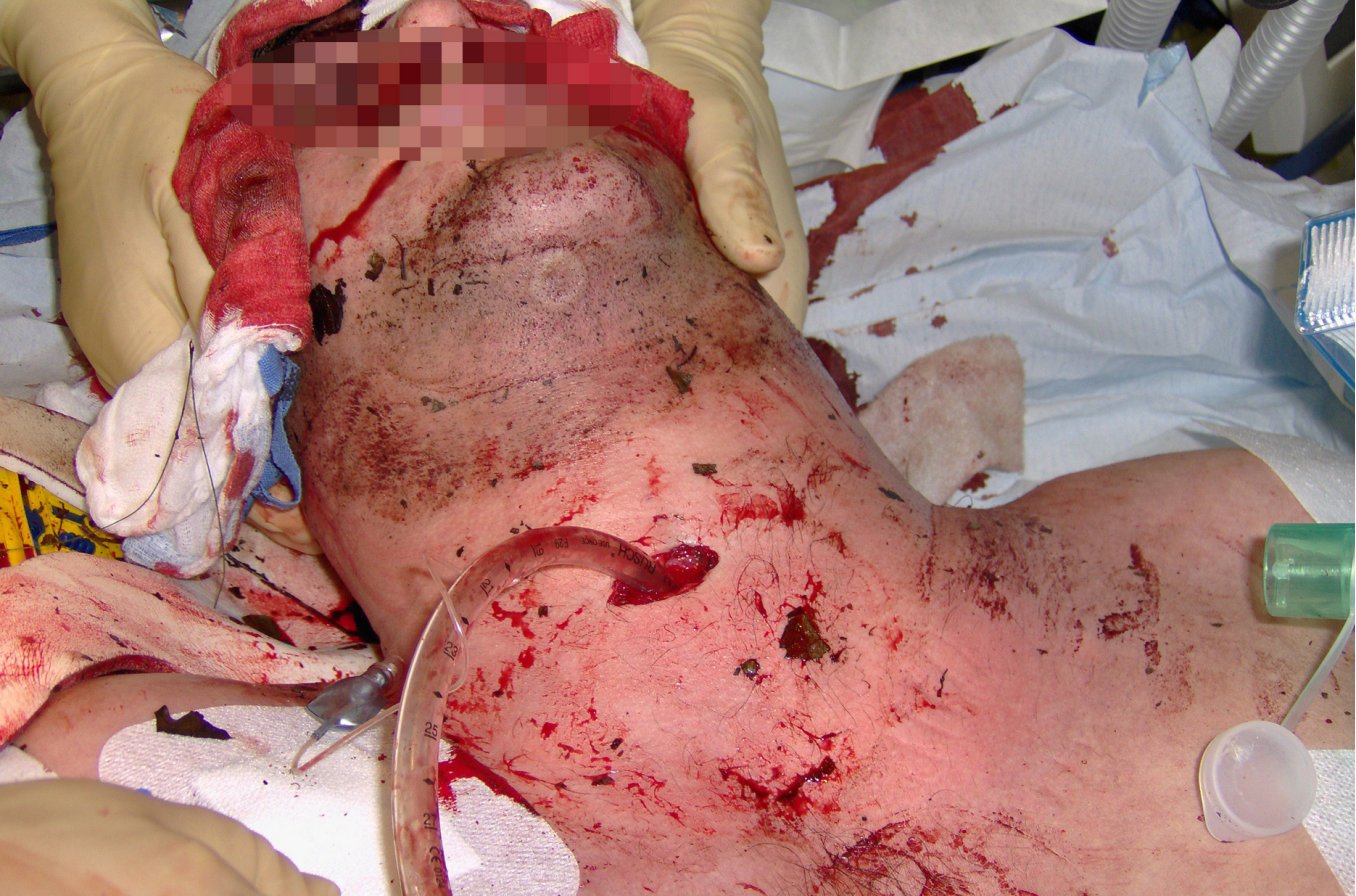
**Massive swelling on the left cervical side after rupture of the vertebral artery**. On scene tracheotomy was performed after endotracheal intubation was unsuccessfully attempted. Coniotomy was not performed due to laryngeal fracture.

We report the case of a 33-year-old Caucasian man who was involved in a quad bike accident in a remote area. After losing control of his vehicle he fell down a 7-meter scarp. Because of cardiopulmonary arrest, his father started mouth-to-mouth resuscitation and cardiac massage on scene. At the time of arrival of a paramedic-staffed ambulance, gasping accompanied with a massive cervical swelling on the left side was detected. Since endotracheal intubation was unsuccessfully attempted due to the severe cervical swelling as well as oral bleeding, the airway was secured with a combitube. He was transported to a level-1 trauma center by a rescue helicopter. For airway protection, an emergency tracheotomy using a 7.5 Fr tube was performed on scene since the larynx could not be palpated for a coniotomy (Figure 1). Chest tubes were inserted because breath sounds were diminished bilaterally.

**Figure 2:**
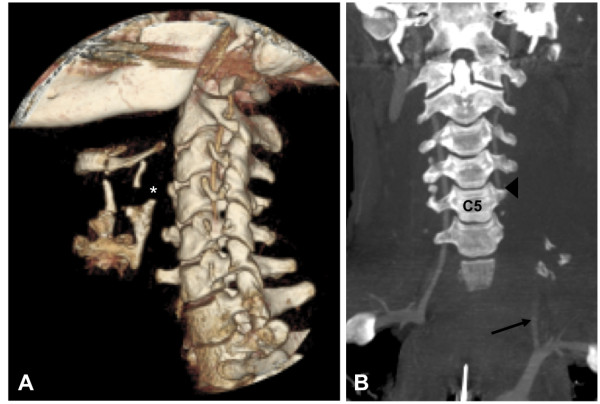
Figure A and B (a) 3D volume rendered (VR) image with fracture of the thyroid cartilage with dislocation of the superior horn on the left side (*). **(b) **coronal maximum intensity projection of the cervical spine (CT angiography scan after intravenous injection of contrast agent). Proximal abruption of the left vertebral artery (arrow) and retrograde filling (arrowhead) at the level of C5.

At the time of presentation at our trauma center, he was hemodynamically unstable in spite of volume resuscitation and administration of vasopressors. Computed tomography (CT) revealed severe injuries limited to the craniocervical region. Extensive bleeding in the mesencephalic as well as pontine region and a stable fracture of the arch of the seventh cervical vertebra and fractures of the transverse processes of C5-C7 with involvement of the lateral wall of the transverse foramen was detected. An abort of the left vertebral artery signal at the first thoracic vertebrae with massive hemorrhage was also present (Figure 2). Additionally, he had a fracture of the left thyroid cartilage and intracerebral hemorrhage. Further evaluation of the CT scan showed retrograde filling of the left vertebral artery at C5 distal of the described abort. He was hemodynamically stabilized after transfusion of six packed red blood cell units and 12 units of fresh frozen plasma. After 24 hours, an additional cranial CT scan was performed and revealed unchanged intracranial bleeding combined with moderate intracranial swelling without any signs of incarceration of the brain stem. Evaluation of the vascular status confirmed the initial finding of retrograde filling of the left vertebral artery up to the abort at the suspected rupture site. Based on these findings he was treated with 10,000IU heparin per day. After a prolonged weaning period, he was transferred to a rehabilitation center specializing in neurological disorders. At the time of discharge, he was aware and moved all extremities on command.

## Discussion

### Blunt vertebral artery injuries

Blunt vertebral artery injuries represent a rare entity but the incidence has increased due to aggressive screening protocols [2]. While digital subtraction angiography is traditionally accepted as the gold standard, computed tomographic angiography is widely used due to its high accuracy. In early case reports, these injuries were only detected by neurological deficits defining the laterality of the cerebrovascular injury. Three mechanisms have been described for blunt cerebrovascular injuries (BCVI): extreme hyperextension and rotation [3]; facet joint dislocation or transverse foramen fracture [4]; and a direct blow to the vessel site [5].

Depending on the origin of the injury, vertebral artery injury may present with intimal disruption (leading to dissection, near-occlusion or occlusion), thrombosis or transection. Most BCVI occur in the vertebral canal in which the vertebral artery is relatively fixed. In patients with BCVI, mortality rates of approximately 25% and permanent severe neurological deficits up to 60% have been reported [6]. The time to diagnosis is extremely variable and correlates with survival [3]. Several screening protocols for patients in which BCVI was suspected have been developed. Helical computed tomographic angiography (CTA) as performed in our patient is the gold standard for the diagnosis of BCVI although no prospective data are available comparing CTA with digital subtraction angiography.

Treatment mostly consists of anti-thrombotic therapy to reduce the risk of embolic complications. This approach has been shown to reduce neurological deficits in symptomatic patients and prevents the development of neurological deficits in asymptomatic patients [6]. However, neuroradiological intervention was used successfully to treat hemorrhagic VAI. Systemic anticoagulation with heparin is the preferred treatment for mild ischemia. Additional relevant injuries, especially intracranial bleeding, need to be considered when anticoagulation therapy is initiated. Due to more aggressive diagnostic algorithms treatment of BCVI can be initiated earlier and BCVI-related neurological impairment as well as mortality has decreased [3].

### Laryngeal fractures

Fractures of the larynx are extremely uncommon. The larynx is well protected by bony structures (that is, the mandible, sternum and cervical spine) and is mobile and therefore rarely injured. The clinical diagnosis may be difficult after blunt trauma but a high level of awareness is necessary since swelling of the unprotected airway as a critical consequence may occur not only immediately after trauma but also after several hours [7].

Diagnosis is made based on clinical findings (for example, hoarseness, laryngeal pain, aphonia, asymmetry, bleeding and subcutaneous emphysema) in the laryngeal area. CT is recommended to evaluate the extent of laryngeal fractures [8].

In a case series, 61% of 33 patients were treated non-operatively with predominantly good results regarding voice and airway [9]. In more severe cases, fracture should be stabilized with titanium nets or mini-plates.

## Conclusions

We describe a new entity after a quad accident with a rare case of vertebral artery injury and a laryngeal fracture. For vertebral artery injuries, early CT scanning and frequent reassessments are recommended. Most patients can be treated with anti-coagulants. The most important step in diagnosing a laryngeal fracture is the physician's awareness and appropriate clinical examination. Management of laryngeal fractures mostly consists of conservative treatment.

## Consent

Written informed consent was obtained from the patient for publication of this case report and accompanying images. A copy of the written consent is available for review by the Editor-in-Chief of this journal.

## Competing interests

The authors declare that they have no competing interests.

## Authors' contributions

MF and CH analyzed and interpreted the patient data and were major contributors in writing the manuscript. KIR performed radiographs and was a major contributor in writing the manuscript. CK and FH have been involved in revising the manuscript critically for important intellectual content. All authors read and approved the final manuscript.
